# Artefacts, practices and pedagogies: teaching writing in English in the NAPLAN era

**DOI:** 10.1007/s13384-020-00416-6

**Published:** 2021-01-27

**Authors:** Susanne Gannon, Jennifer Dove

**Affiliations:** Centre for Educational Research, School of Education, Western Sydney University, Locked Bag 1797, Penrith DC, NSW 2751 Australia

**Keywords:** Writing pedagogy, Secondary English, NAPLAN, Instructional artefacts, Case study at a distance

## Abstract

In secondary schools, English teachers are often made responsible for writing results in national testing. Yet there have been few studies that focussed on this key group, or on how pedagogical practices have been impacted in the teaching of writing in their classrooms. This study investigated practices of English teachers in four secondary schools across different states, systems and regions. It developed a novel method of case study at a distance that required no classroom presence or school visits for the researchers and allowed a multi-sited and geographically dispersed design. Teachers were invited to select classroom artefacts pertaining to the teaching of writing in their English classes, compile individualised e-portfolios and reflect on these items in writing and in digitally conducted interviews, as well as elaborating on their broader philosophies and feelings about the teaching of writing. Despite and sometimes because of NAPLAN, these teachers held strong views on explicit teaching of elements of writing, but approached these in different ways. The artefacts that they created animated their teaching practices, connected them to their students and their subject, suggested both the pressure of externally driven homogenising approaches to writing and the creative individualised responses of skilled teachers within their unique contexts. In addition to providing granular detail about pedagogical practices in the teaching of writing in the NAPLAN era, the contribution of this paper lies in its methodological adaptation of case study at a distance through teacher-curated artefact portfolios that enabled a deep dive into individual teachers’ practices.

## Introduction

As we were writing this paper about research conducted in 2019, the COVID-19 pandemic reshaped our lives, our work and every aspect of education. The imperative to keep communities safe and well led to the cancellation of NAPLAN for 2020, in what would have been its 12th year, and to a shift to online learning for schools in order to maintain physical distancing. For researchers, it has prompted innovative rethinking of research practices (Lupton 2020). Perhaps, in addition to contributing to understanding current instructional strategies in teaching writing, this paper will contribute methodologically with its approach to case studies at a distance.

Writing results, as measured by NAPLAN, are of concern to all sectors and stakeholders. A continuing decline in writing at secondary level has been reported in most years and most states. Whilst there have been numerous investigations of NAPLAN (Polesel et al. 2014; Rose et al. 2018; Swain et al. 2018), only a few have focussed on writing (Caldwell and White 2017; Spina 2017; Thomas 2019). Fewer have focussed on secondary schools, where the initial cross-disciplinary intent of NAPLAN tended to obscure the practices of English teachers (Carter et al. 2018; Frawley and McLean Davies 2015; Simpson Reeves et al. 2018). Yet this group is most likely to be positioned as experts in teaching writing, most often charged with leading improvement, and most often blamed when this does not happen (Gannon 2019). In 2016, NAPLAN alignment with national curriculum in English, rather than a broader focus on literacy, was announced by the Australian Curriculum Assessment and Reporting Authority (ACARA 2016). However, though pointing more directly at English teachers, this has not ameliorated concerns or improved results.

The crisis in writing is well established and tenacious. Beyond media accounts, recent statistical analyses have suggested “rapid decline” (Thomas 2019, p. 1) and “negative accelerating change” (Wyatt-Smith and Jackson 2016, p. 233). Concerns have been articulated in recent state-based NAPLAN reviews. In Queensland, Cumming et al. (2018) recommended further exploration of teaching and assessment of writing performance. An Australian Writing Survey (AWS) developed by Wyatt-Smith and Jackson indicated that secondary teachers felt underprepared for teaching writing (Cumming et al. 2018). Focus groups found widespread perceptions that NAPLAN writing tasks were opposed to what were perceived to be good pedagogical practices in teaching writing, such as developing skills in planning, drafting and editing, and that NAPLAN had directly “diminished the quality of student writing” (Cumming et al. 2018, p. 81). A recent NAPLAN review commissioned by state governments singled out the writing task for extended critique with key findings identifying “a sustained thread of dissatisfaction” amongst stakeholders, confirming that the writing task has had “unintended effects on how writing is taught,” that it produces “formulaic” writing, and that its failures seem to be particularly evident in secondary schools (McGaw et al. 2020, p. 83). Whilst the study mentions “rich writing pedagogy”(McGaw et al. 2020, p. 87), this is not developed in depth. For insights into writing pedagogies, including professional judgements and practices of teachers in situ in Australian schools, it is necessary to consider more closely, and in collaboration with teachers, what happens in their classrooms when they teach.

In this paper, we focus on these intricacies of classroom practice. The study was designed by Susanne as a sequential mixed-method study that could both report teachers’ impressions and experiences of NAPLAN’s impact on the teaching of writing on a broad scale and take a deeper dive into a small number of classrooms and the pedagogies of writing that were in evidence. The pilot was undertaken in two states, Queensland and Tasmania, and focussed on writing in secondary English for years 7–10. Phase 1 comprised an online survey opened for 1 month in late 2018 which surveyed teachers’ beliefs, practices and experiences in teaching writing (Gannon 2019). Phase 2 was a qualitative multi-sited case study undertaken in four schools throughout 2019. State English Teachers’ Associations circulated the Phase 1 survey to members and suggested potential case studies for Phase 2. In two schools, data collection involved several teachers, including the head of English. Ethical agreement was secured from participants, Principals and state Departments of Education. Jennifer joined the project after data collection was complete, bringing her experience as a secondary English teacher and researcher of writing to assist with data analysis and case construction (Dove 2018; Dove and Gannon 2017).

## Artefacts as method

Whilst many studies of writing draw on student data (writing samples, test results), and teacher interviews and classroom observations are standard qualitative methods, few studies approach writing through artefacts. Narrative approaches to English teachers’ practice provide rich and situated accounts of practice, and such narratives could be considered a mode of classroom artefact (Parr and Bulfin 2015; Parr et al. 2015). However, in this study, we take up classroom artefacts more precisely as those pedagogical or instructional artefacts—concrete material objects—that are deployed or created in pedagogical events in classrooms. In this orientation, our research gestures to a turn towards materiality more broadly where ‘things’, as they entangle with bodies and discourses, demand analytical attention (Heimans 2015).

In previous research on the teaching of writing by exemplary teachers, teacher interviews have been insightful and generative but discussions have tended to be untethered from the material minutiae of practice (Gannon and Davies 2007). In-class observations are skewed by the gaze of the researcher who is an intruder in the classroom. Although observation tools can guide such work, there are likely still to be omissions. Ideally observation and interview are combined (Gannon 2011, 2014); however, for pragmatic reasons, one or two observed lessons tend to be plucked out of a sequence in a fairly arbitrary fashion to suit the exigencies of time. In research with experienced teachers, the various objects that teachers produce to mediate and materialise pedagogy as practice are less frequently the focus of attention.

This research required a systematic approach and consistent protocol that could accommodate case studies of pedagogical practice across multiple sites, to allow for the pilot to be scaled up if conditions were amenable.^1^ We drew on the artefact-led design of US scholars Borko, Martinez, Stecher and others—variously called “Scoop”, “e-portfolios” and “artefact packages”—developed for large-scale evaluation of middle school Science and Mathematics pedagogies (Borko et al. 2005, 2007; Martinez et al. 2012). Their approach was compatible with the intention to inquire into the minutiae of practice and to foreground the agency of teachers as designers of teaching and learning. Principles that informed their work and ours were that pedagogical artefacts were selected by teachers, from a sequence of lessons, with accompanying written reflections. Modifications were driven by funding, the limits of scale and an interest in maximising opportunities for teachers to elaborate on their pedagogy. Whilst Borko et al. collected artefacts across 5–7 days of instruction in Maths or Science, we asked teachers to upload artefacts across a sequence of ten lessons in English. Borko et al. (2007) asked teachers to select three categories of artefact in every lesson: materials produced before class (e.g. tasks, rubrics), materials generated during class (e.g. board notes) and materials generated outside class (e.g. homework). Our study offered these as examples, but asked teachers to choose one artefact each lesson of any of these types that reflected their approach to teaching writing. Reflecting the times, Borko et al. (2007) initially used hard copy binders, and later e-portfolios in a purpose-built online portal (Martinez et al. 2012). For this study, password-protected folders were created in Cloudstor, the online storage service owned by Australian and New Zealand universities. Each teacher had their own folder, visible only to them and the researcher. Folders contained instructions on uploading artefacts and a short video was also produced by Susanne to demonstrate the process. Like Borko et al., teachers were asked to complete a daily reflection sheet for each artefact explaining their intentions, sequencing of learning in the lesson and so on. Access to individual folders expired 6 weeks after upload. Given the shifts in technology since the work of Borko et al., whilst our participants included tasks, rubrics and homework amongst their artefacts, they also used smartphones to photograph whiteboards, student notebooks, classroom displays and pedagogical props, and they deployed newer technologies in their teaching such as Powerpoint and class-specific Flipgrid sites.

Our interest in attending to artefacts acknowledges the heterogeneity of practice. Despite equivalent teacher training, professional networks and common curriculum, we did not expect to see identical practice in the teaching of writing. For Borko and colleagues, the large-scale collection of artefacts facilitated validation of a set of ten dimensions of instructional practice for Science and Mathematics, aligned with national curriculum standards. They developed a scoring sheet and trained subject specialists to code the artefacts. Validation of their instrument against the national standards was their focus, and they worked with large numbers of teachers in multiple locations (Martinez et al. 2012). This small-scale study had a different intention. Rather than testing or validating a predetermined instrument or generalising and standardising practice across a large number of cases, we were interested in the nuances and variations of practice. That is, what do we find when teachers of writing in secondary English represent and talk about their practice?

Approximately 2 weeks after the collection of artefacts across 10 consecutive lessons, individual semi-structured interviews were held via Zoom with participating teachers. Zoom allowed us to screen-share each artefact and discuss them one at a time during the interview. Prompts were shared by email before the interview, so that the teachers had time to think about their responses. In all, eight interviews were held in four schools, with seven of these in a school office or staffroom. In addition to more detailed discussion of the work done by each artefact in the classroom, interview prompts led to discussions of teachers’ philosophies and approaches to teaching writing, perceptions of a ‘good class’ and goals for their students.

Importantly, in this qualitative phase of the study NAPLAN was not specifically named in the interview prompts. However, all teachers had signed up for research investigating writing pedagogies “in the NAPLAN era” and it arose in all the interviews. Open-ended questions included: “Are there particular contexts that impact on the teaching of writing?”, “What else would you like to add about how you approach the teaching of writing? Or about teaching writing more broadly in English?” These questions were designed to invite teachers to comment on NAPLAN amongst other influences, but they did not require them to privilege NAPLAN as *the* most influential factor. They were not directed as to how they were to position NAPLAN. Where NAPLAN was raised, as it was by all teachers, the interviewer followed the lead offered by the teacher. For example, when a newspaper headline about NAPLAN was mentioned, the interviewer followed with “Do you use that [NAPLAN] as evidence of how well you’re going?”. In another interview, the interviewer asked a teacher to expand on a response that included the comment “we kind of plan around NAPLAN a little bit as well. That’s just the kind of the expectation”. Other teachers responded directly, such as in this exchange with an early career teacher:

- Interviewer: Are there other particular contexts that impact on the teaching of writing in your location?
- Teacher: NAPLAN.

The teacher elaborated that students coming from primary school have had persuasive and narrative writing structures “hammered into their heads” so their writing looks “exactly what a NAPLAN script would be”. We have added in these detailed examples to provide more insight for readers into how we proceeded in this study, allowing NAPLAN to emerge somewhat obliquely and inviting teachers to elaborate the pedagogies that were evident in the classroom artefacts that they chose to share. We return to the NAPLAN comments from the interviews in the final section of the paper.

## Case study sites and protocols

Four pilot case studies were conducted with two schools each in Queensland and Tasmania. One state school and one Catholic school were included in each state. Two schools were regional and two were located in state capitals. Institutional ethical approval meant that systems could not be compared; therefore, in this paper, we do not identify schooling systems or states when discussing case studies. Teachers chose one class from year 7 through to year 10 to focus on for artefact collection across 10 consecutive lessons. Given that writing tends to be approached as developmental, this is the order in which case studies are presented in this paper.

Teacher interviews were crucial for the study. As always in research that aims to respect the autonomy of teachers, the design was modified in situ and in negotiation with participants. In two schools, Susanne conducted interviews with a single participant teacher, whilst in the two other schools separate interviews were held with the head of English as well as participating teacher. In all, eight interviews ranging from 36 to 72 min were held for the project. One school selected two participating teachers because they co-plan lessons and units of work and often team teach. In another school, the head of English and classroom teacher divided the task and uploaded five artefacts each. Teachers ranged in experience from a recent graduate through to more than a decade. Case studies took place when it was most convenient for teachers and according to the timing of ethics approvals in each state.

Artefacts were the primary focus of the case study in each school, and the teacher interviews followed in order to tease out the pedagogical impacts of the selected artefacts and to explore broader issues relating to the teaching of writing. It was in this latter section of the interview that NAPLAN tended to be addressed. The first artefacts and written reflections were uploaded in early February 2019 in one school and extended through to mid-December 2019 with the fourth school. No artefacts were uploaded in the months of April or May when direct emphasis is on NAPLAN in schools. The final interview took place on the last day of the school year. By the end of the year, 47 artefacts and reflections had been uploaded into Cloudstor e-portfolios. Although teachers were asked to focus on a single class at a single year level, only two of the teachers did this, and other collections included artefacts from several year levels. We have endeavoured to construct one case at each year level, from each school, to ensure an even spread across participating schools and teachers. These are reported in chronological order from year 7 through to year 10 and are not intended to be equivalent or comparable in any direct way. However, they are each meant to be useful anchors for elaborations of practice. They are artefact-led in that each case study samples artefacts and explores how those artefacts lead teachers to talk about writing pedagogy.

It is important to note, however, that this paper discusses around only 10, or around 20%, of the much larger collection of instructional artefacts. We selected artefacts in terms of their inherent interest, their variety and the scope they provided for teachers to articulate writing pedagogies. Some of our decisions were pragmatic in that a single artefact may be too big to do justice to in this paper (e.g. a 56 slide PowerPoint file that encompassed all activities and resources across a week of lessons), or may be a stimulus text from elsewhere (e.g. newspaper article, poem, extract from a novel), or where a school was identifiable on the artefact (e.g. school logo on assessment task or rubric). We were most interested in teacher produced artefacts and in artefacts that mediated learning, including scaffolds, tools, displays and in-class documentation on whiteboards, or from group or individual activities. Our selections were made so that each of the four schools provided a coherent account of an aspect of writing pedagogy that the teachers elaborated in their interviews and written reflections. That meant omitting some artefacts from our discussion where we could not discern a connection to writing pedagogies. However, given the nature of the subject, we note that reading and writing are integrated textual practices in many lessons. During the process of developing this paper, we also created descriptive case studies^2^ for each school which have been checked and verified by the participating teachers.

## Year seven

This case study focusses on creative and expressive writing. Most of the uploaded artefacts were images taken in the classroom, with two word documents (assessment task, rubric).

**Fig. 1:**
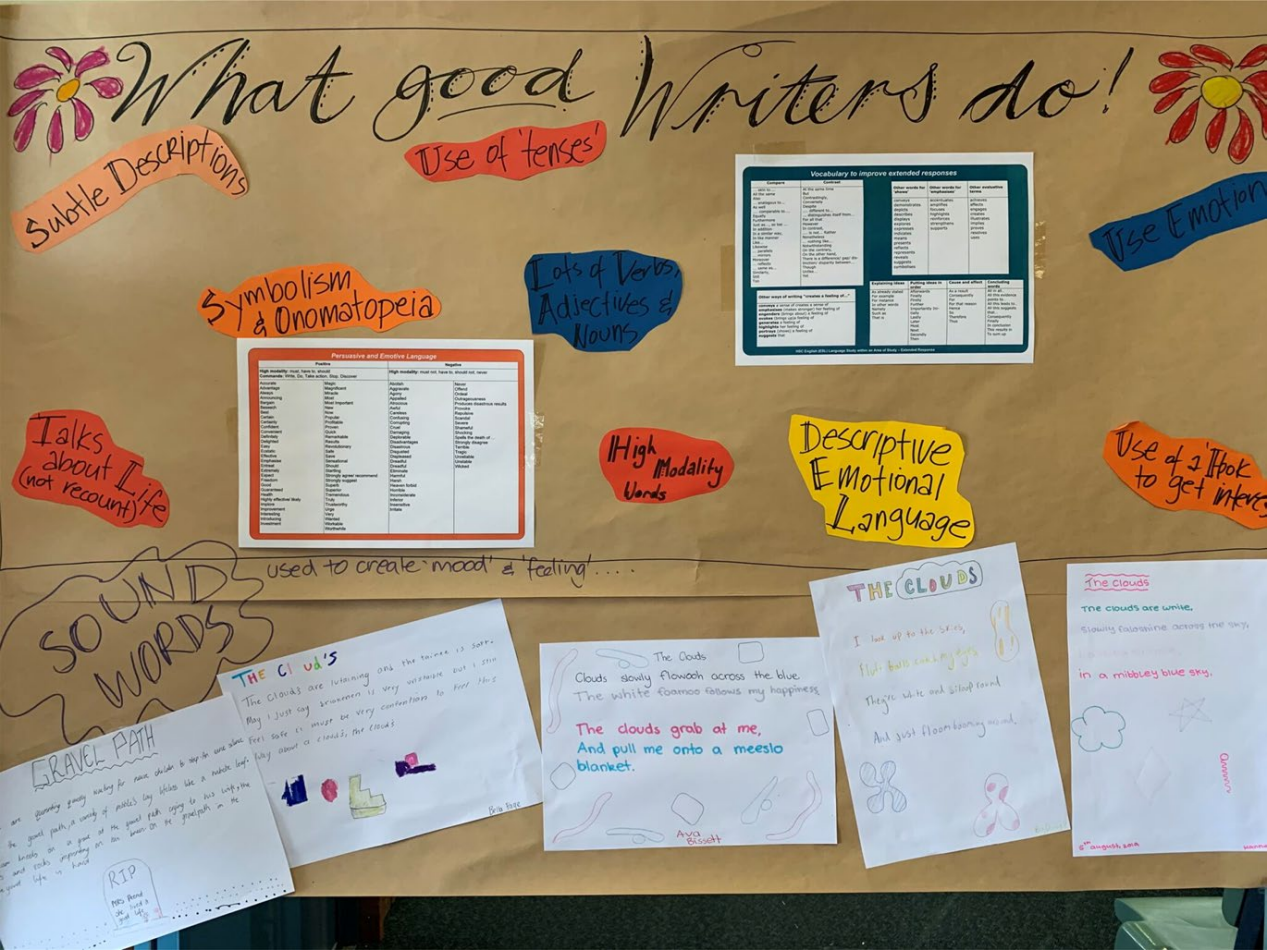
Anchor Chart. What good writers do!

The first artefact uploaded was an image of the display board on the back wall of the classroom, suggesting its significance in defining this teacher’s approach to writing (Fig. 1). The public iteration of “What good writers do!” is a dynamic fixture in this classroom. This is not a glossy commercial poster but an artisanal handmade object, with a brown paper background, beautiful calligraphy, hand-drawn pink and yellow daisies and bubbles of bright colour identifying language features: “Descriptive emotional language”, “Use of a ‘hook’ to get interest”, “High modality words”, “Subtle descriptions”, “Use of emotion”, “Lots of verbs, adjectives and nouns” and “Symbolism and onomatopoeia”. Additional items are added through the year. For example, there are lists contrasting “positive” and “negative” words and “high and low modality” words. These are vocabulary resources that students are expected to draw on. The bottom third of the display comprises five illustrated examples of student writing: one called “The gravel path” and four entitled “The Clouds”.

This teacher ensures that writing is not allowed to become “stagnant and very rigid”. She maximises opportunities for practicing writing in low stakes contexts and emphasises pleasure and play. Students should “just play with words, play with language features, even if they don’t know what they are”. However, the display suggests that she is equipping students with an appropriate metalanguage. This is serious play within the disciplinary knowledge of subject English. This teacher emphasises how important it is to allow students to develop a writerly style or voice of their own. Students need “to play with their own way of writing rather than say you have to write this, this, this and it has to look like this, this, this”.

The student texts attached to the display exemplify this play with language. During a vocabulary-focussed unit, students started talking about “hard” and “soft” words, referring to both their aural qualities and what they signified in the world. This was a “totally random thing” rather than a deliberately planned episode within a lesson. When asked for examples of “soft” words, they offered “dandelion” and “pillow”, and for “hard words” they offered “tick”, “kettle” and (tellingly) “rubric”. Then students were asked to use the random words they had generated to create “a poem for a cloud and a poem for a rock”. They were not required to make sense in a coherent way, but rather to play with the audio qualities of language and explore how vocabulary can affect meaning. The poem called “The gravel path” was a variation on “a poem for a rock”. Poetic intensity is achieved through metaphor and alliteration, and by contrasting naïve children with a grieving widower, the young writer has artfully evoked a poignant scene of a gravel path leading to a graveside.

**Fig. 2:**
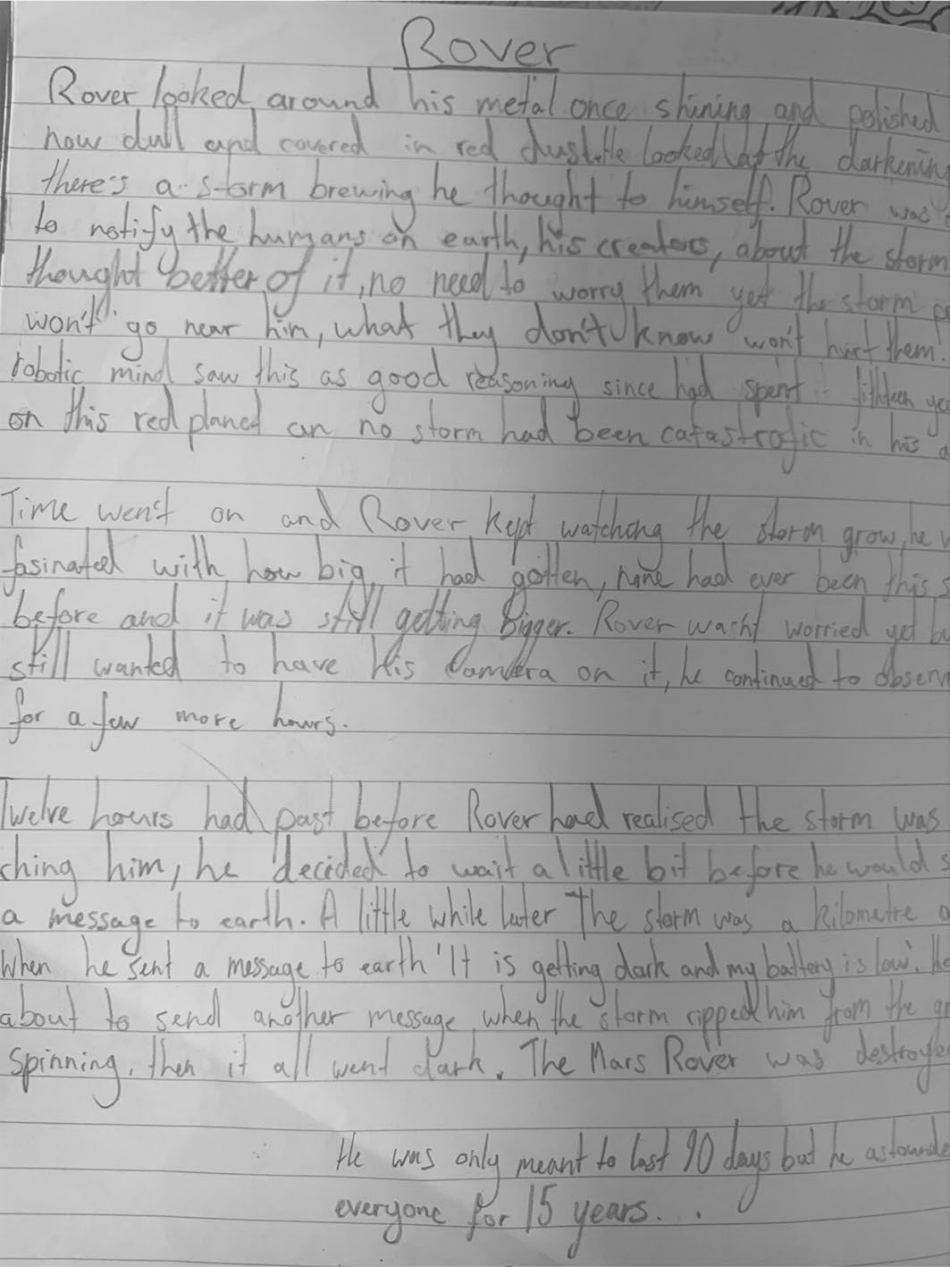
Rover. Student work sample

In contrast to the public display of “What good writers do!”, the Writer’s Notebook is a private affair (Fig. 2). This is not unique to this class or teacher, but characterises how writing is approached across this school. In later years, there is an elective Writers Workshop, and writing competitions operate like sports competitions as students accrue points for their school house. The two artefacts from the Writers’ Notebook are images of a page of student writing and a handout: “Rules of the Writers’ Notebook”:

1. You will respond to prompts and explore your own ideas.
2. You may extend these ideas beyond the prompt and form them into a larger piece of work.
3. All drafts, revisions, editing and final copy occur outside of the notebook (pad paper or word processing).
4. The teacher will not mark your work, just monitor.
5. Have fun writing! (bolded)

**Fig. 3:**
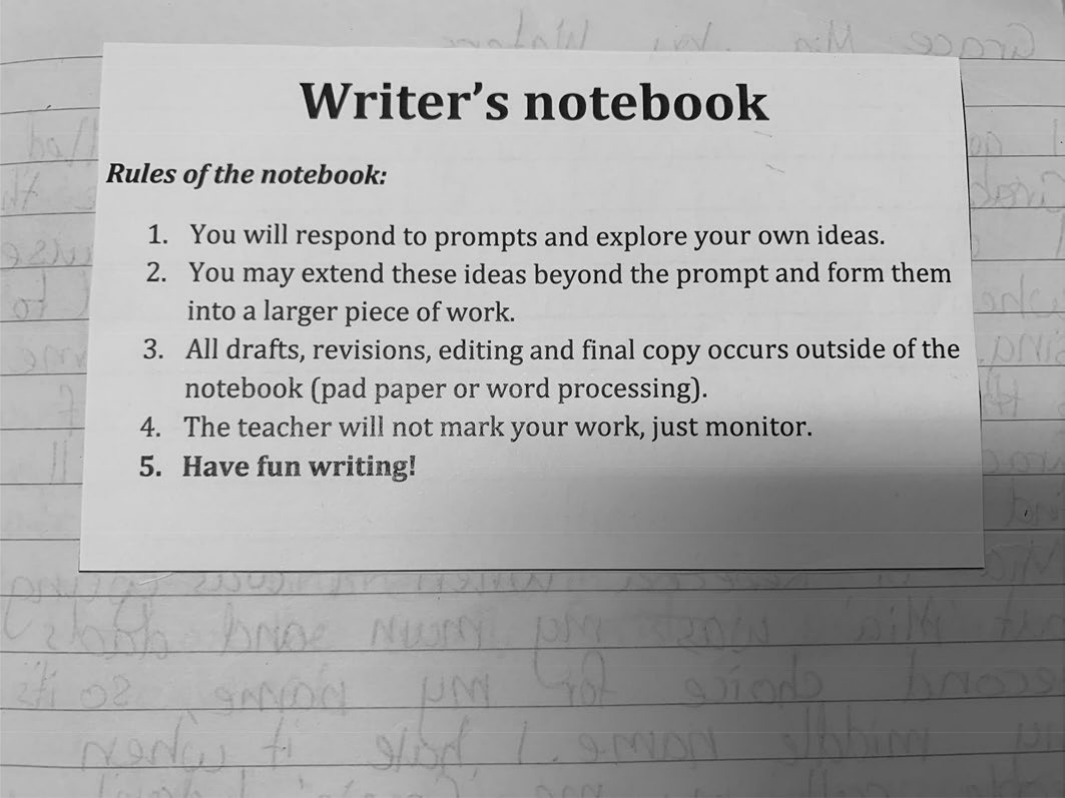
Writer’s notebook

The story, “Rover”, begins: “Rover looked around, his metal once shiny and polished, now dull and covered in red dust….” (Fig. 3). The prompt was an image and an invitation to students to write a space story. “Rover” displays a sophisticated grasp of personification, an essential textual concept for literary writing, as the student imaginatively inhabits and animates the robotic space monitor of NASA’s Mars mission. Writer’s Notebook has a dedicated weekly timetable slot. It offers “low stakes” conditions, aiming to “improve vocabulary, writing stamina, writing confidence …without the pressure of assessment”. Each class begins with a prompt, an example and/or a mini-lesson on a textual concept or language feature, followed by 10 min of quiet uninterrupted writing. The teacher reflected that across the year, the notebook had helped increase confidence, writing quantity and quality, and willingness to share in small groups. However, point 2 of the “rules”, extending first drafts into “more formal pieces of writing”, will be the focus in future iterations of the initiative.

## Year eight

This case study focusses on highly scaffolded approaches to textual analysis. Artefacts included instructional PowerPoints, tasks, text excerpts, worksheets and handouts. They demonstrate how students are guided to develop skills for close reading and writing about texts.

**Fig. 4:**
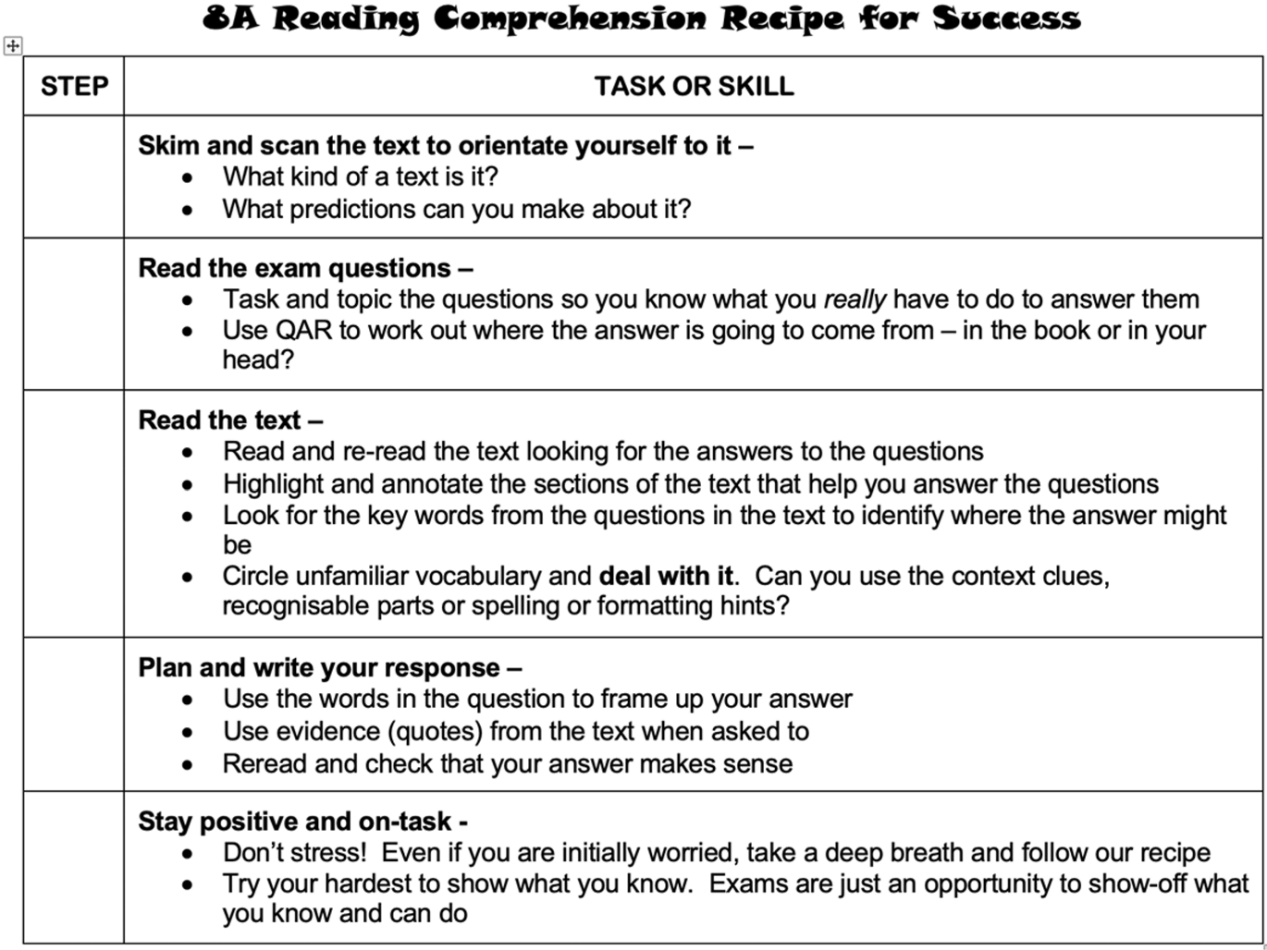
Reading Comprehension Recipe for Success

The A5 “Reading Comprehension Recipe for Success” (Fig. 4) offers five steps for answering questions about a text, which students are to follow and tick off. Students are directed to “Skim and scan the text to orientate” themselves and questions are provided to assist with that orientation process (“What kind of text is it? What predictions can you make about it?”). An upcoming formative reading comprehension task is foreshadowed in the next step, “Read the exam question” with students directed to “Task and topic the questions”. This is a school-wide process used for all tasks to ensure students understand task requirements and is more commonly known as “tapping out” the task (Topic, Task, Audience, Purpose). Students are reminded to use QAR (Question-Answer Relationships) “to work out where the answer is going to come from—in the book or in your head?”. The third step is to “Read the text” and re-read, highlighting “sections of the text that help you answer the questions” and identifying key words and unfamiliar vocabulary. For the latter, students are told to “Deal with it” using “context clues, recognisable parts or spelling or formatting hints”. After reading, the fourth step in the Recipe for Success is for students to “Plan and write your response” using words from the question and evidence (quotes) before rereading their answer to check that it “makes sense”. Finally, students are encouraged to “Stay positive and on-task: Don’t stress! Even if you are initially worried, take a deep breath and follow our recipe; Try your hardest to show what you know. Exams are just an opportunity to show off what you know and can do”.

In the lesson sequence, students used these familiar metacognitive approaches in their study of representations of young people in newspaper articles and fiction. The teacher describes how the school follows an approach that promotes explicit teaching of reading skills through authentic reading tasks and an explicit writing process: “I’m always trying to get them to revise and edit their work and… I think also that it’s got a pretty close relationship to reading…we always tell the kids [that] those who read end up being the best writers”.

**Fig. 5:**
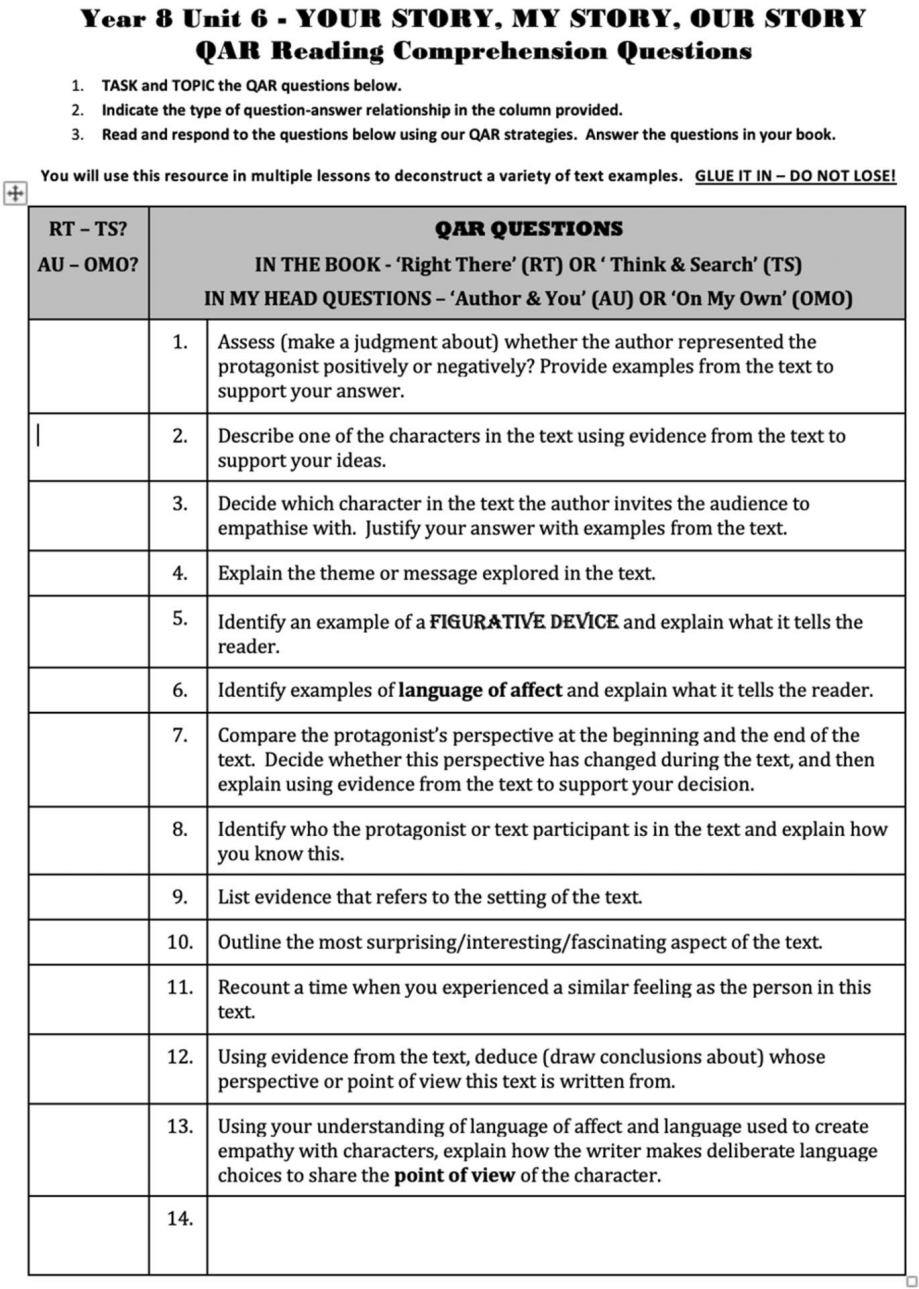
QAR Reading Comprehension Questions

The next artefact uploaded was the QAR (Question-Answer Relationships) reading comprehension guide (Fig. 5) that is used in the Unit “Your Story, My Story, Our Story”. The QAR questions guide students to make decisions about where to find information: “IN THE BOOK—‘Right There’ (RT) OR ‘Think & Search’ (TS) or ‘IN MY HEAD QUESTIONS’—‘Author & You’ (AU) OR ‘On My Own’ (OMO)”. The 13 questions reflect common strategies for teaching students inference and are designed to be used with various texts so students are to “GLUE IT IN—DO NOT LOSE!” Although the questions direct students to look for information about elements including character, theme, figurative language, setting and point of view, this artefact is less about teaching textual analysis than explicitly teaching the reading strategies that equip students to analyse a text effectively. The tick-a-box or coding approach offers students a sense of accomplishment as they work through the list, recording short answers to the questions in their notebooks. Students are also directed to provide evidence from the text in their responses, from simple instructions such as “List evidence that refers to the setting of the text” to more complex questions: “Compare the protagonist’s perspective at the beginning and the end of the text. Decide whether this perspective has changed during the text, and then explain using evidence from the text to support your decision”.

Reading and viewing comprehension activities that emphasise character and language of affect guide students to identify emotions and articulate the ways an author engages readers.

Whilst providing challenging and engaging texts for the story unit, including *Rabbit-proof Fence* and *Nanberry: Black Brother White*, the teacher expressed an intention to reduce the “cognitive load” of analysis in order to focus on writing and assist students to develop effective writing skills that incorporate the language of affect, judgement and appreciation influenced by systemic functional linguists. The motivation is to show students how to reach outcomes rather than simply demonstrating model texts: “a good lesson for me is where I have set them up and they have written something, they’ve produced something and they’ve learnt something from the experience. They’ve then had the opportunity to… apply something different in a new way. That’s the dream.”

## Year nine

Artefacts for year 9 came from two schools; however, each school only uploaded four items for this year level. Here, we discuss how the TEEL approach to paragraphing was adopted in a unit on *Macbeth*.

**Fig. 6:**
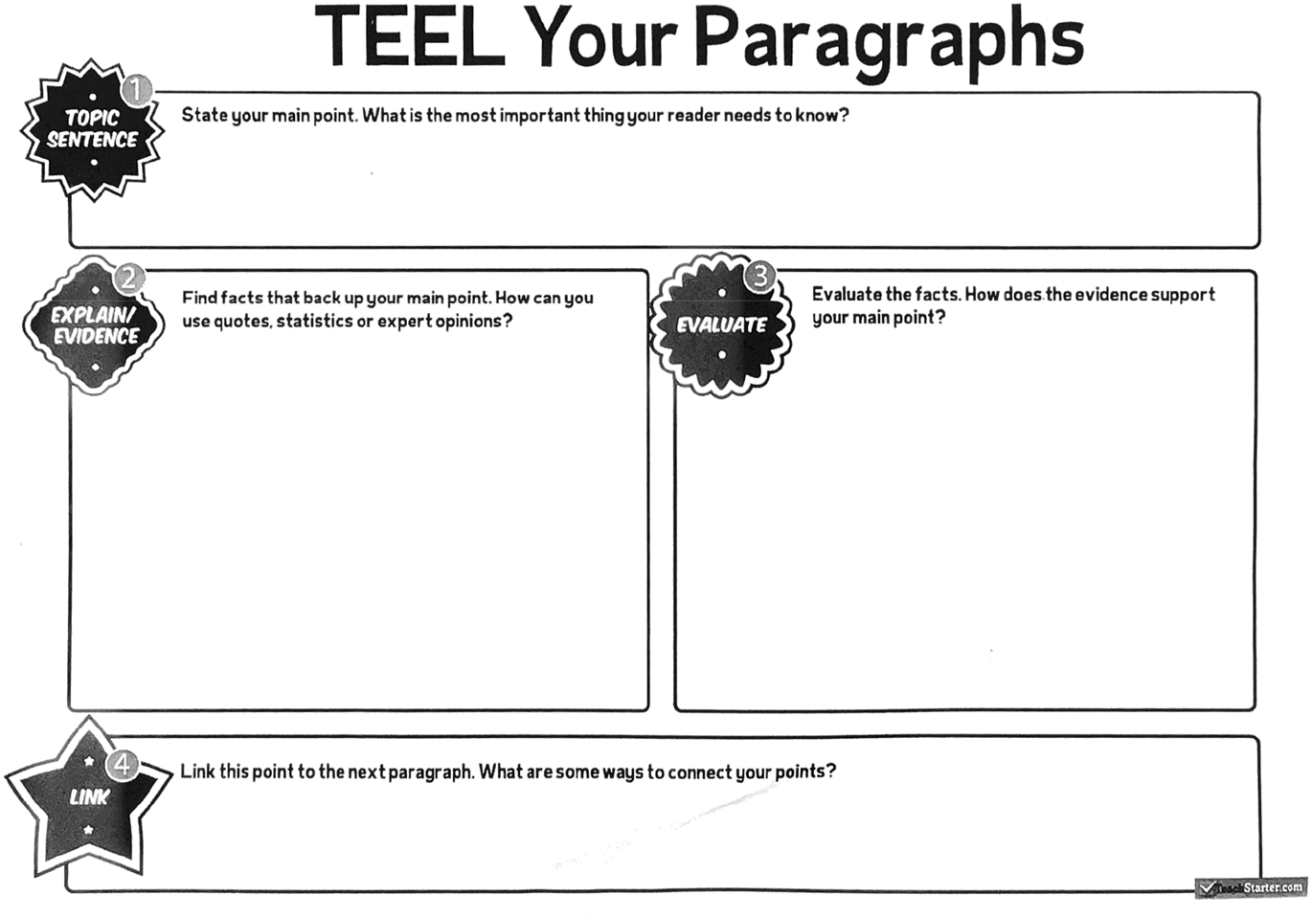
TEEL Your Paragraphs

This handout demonstrates the consistent approach to scaffolding writing taken up at this school. This common version of a paragraph structure uses the acronym TEEL: “1. Topic sentence—State your main point; 2. Explain/evidence—Find facts that back up your main point; 3. Evaluate—How does the evidence support your main point; 4. Link–link this point to the next paragraph” (Fig. 6). The graphic organiser is sourced from a commercial provider, with a logo in the bottom right hand corner (Teachstarter.com). It comprises four unlined boxes in which students can “brainstorm and organise”. It aims to assist them in planning and writing a structured paragraph as they work towards a short essay on *Macbeth* to be written under exam conditions. The minimalist nature of the worksheet, whilst less complex for students to negotiate, led the teachers to a struggle to encourage students to provide more than “one explanation, one bit of evidence… it’s pretty much like E recurring until you decide if you need any more”. Although the TEEL organiser is generic, intended to apply to any argumentative text, in this literary context, “evidence” or “facts” will always be quotes or textual details that support the interpretation being offered. These were seen as skills that would be crucial for students’ success in the upper years of secondary English, and therefore needed to be a focus of explicit teaching in the middle years.

**Fig. 7:**
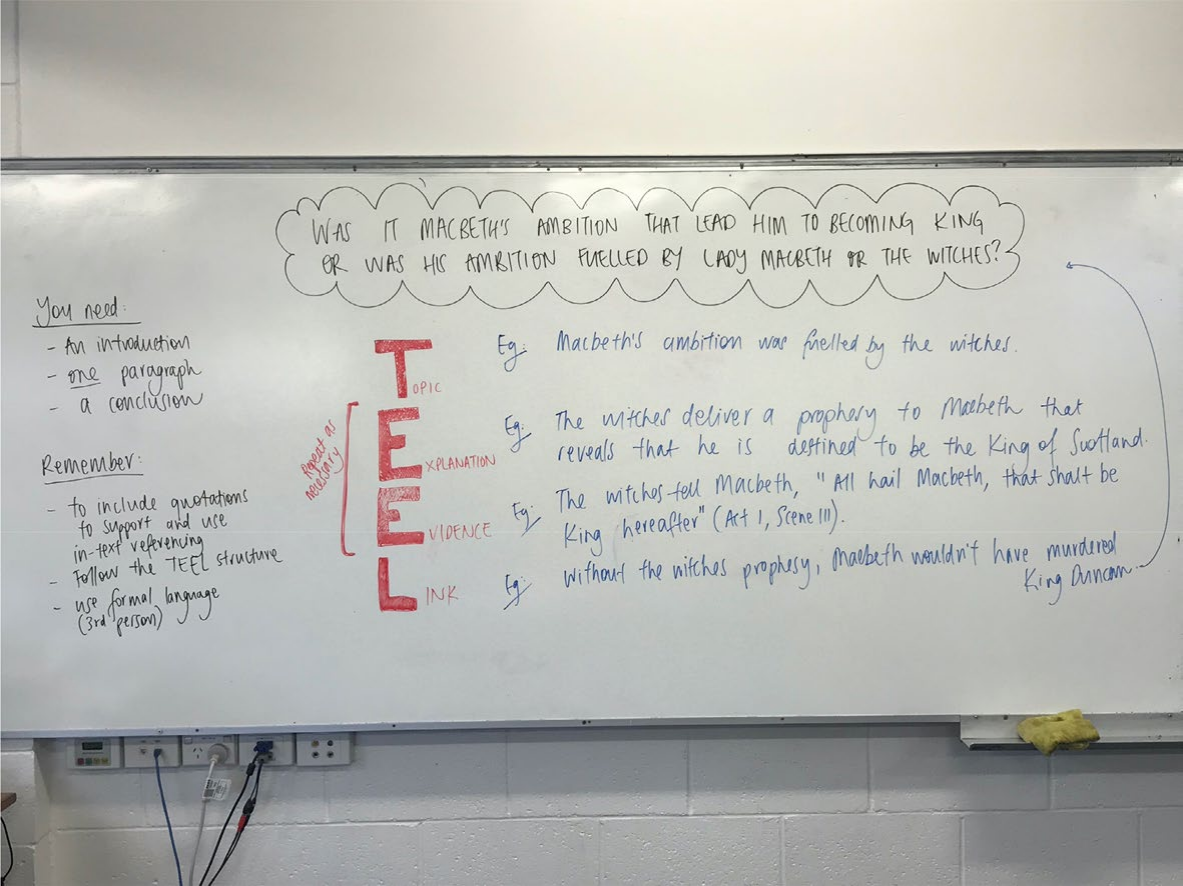
TEEL in Macbeth

The image of TEEL whiteboard notes from one lesson demonstrates how the generic paragraph approach is applied in a specific context through modelling and joint construction (Fig. 7). The class and teacher work together to develop the foundations of a TEEL paragraph answering the question: “Was it Macbeth’s ambition that led him to becoming king or was his ambition fuelled by Lady Macbeth or the witches?”. TEEL is written in red block print down the middle of the whiteboard, with each letter of the acronym unpacked like an acrostic. Examples of sentences and evidentiary quotes are provided for each of: **T**opic/**E**xplanation/**E**vidence/**L**ink. Students then write their own version of the paragraph in their notebooks, drawing from the whiteboard notes.

Overall, the most challenging part of the process for the teacher is the L in TEEL: “linking it back to the question and linking it forward to the next paragraph”. Whilst the worksheet itself makes no reference to a question, or to specific literary texts, the teacher reflects that the scaffold is a useful resource. It is provided along with the essay question, and students are encouraged to articulate how their quotes “related to the question” and identify “key words in the question” that they can include.

The new head of English had decided to introduce TEEL as a response to students’ underdeveloped analytical skills and tendency to merely retell the story of a studied text without understanding the need to include and evaluate evidence. Teachers were asked to use the graphic organiser in every English class as part of a combined attempt to “embed” a consistent approach to writing. The new head was concerned that “in secondary, people assume that they have all the skills and they know how to write and they don’t actually teach how to do it”. Scaffolds are important “not just for people that struggle” but also those who need assistance with organising their ideas. To reinforce the importance of structure in a literary analysis essay, teachers are using it “across the board. Everyone’s doing TEEL”. Year 9 students have responded favourably: “This is great. We wish we’d had it before”. Asked when students could be “weaned” from the scaffold, the teacher admitted it was hard to say: “I’ve had Grade 10s that would rely on this and then I’ve had some that don’t even need it”. The ideal would be “that they could walk into an exam and write—plan a TEEL paragraph without something like this”. By year 10, in some classes, writing the acronym on the board with a quick reminder to plan using the TEEL approach was deemed sufficient.

The TEEL approach is just one of a range of structured approaches to writing that provide students with a template for persuasive genres of writing. Whether this highly structured approach to writing will continue to be effective in addressing the writing demands of senior English, particularly with new Syllabus genres and requirements, is uncertain.

## Year ten

This case study incorporated clusters of artefacts focussed on a single skill or concept (modality, punctuation for effect). Here, we focus on mastery of metalanguages of grammatical choice and literary style.

**Fig. 8:**
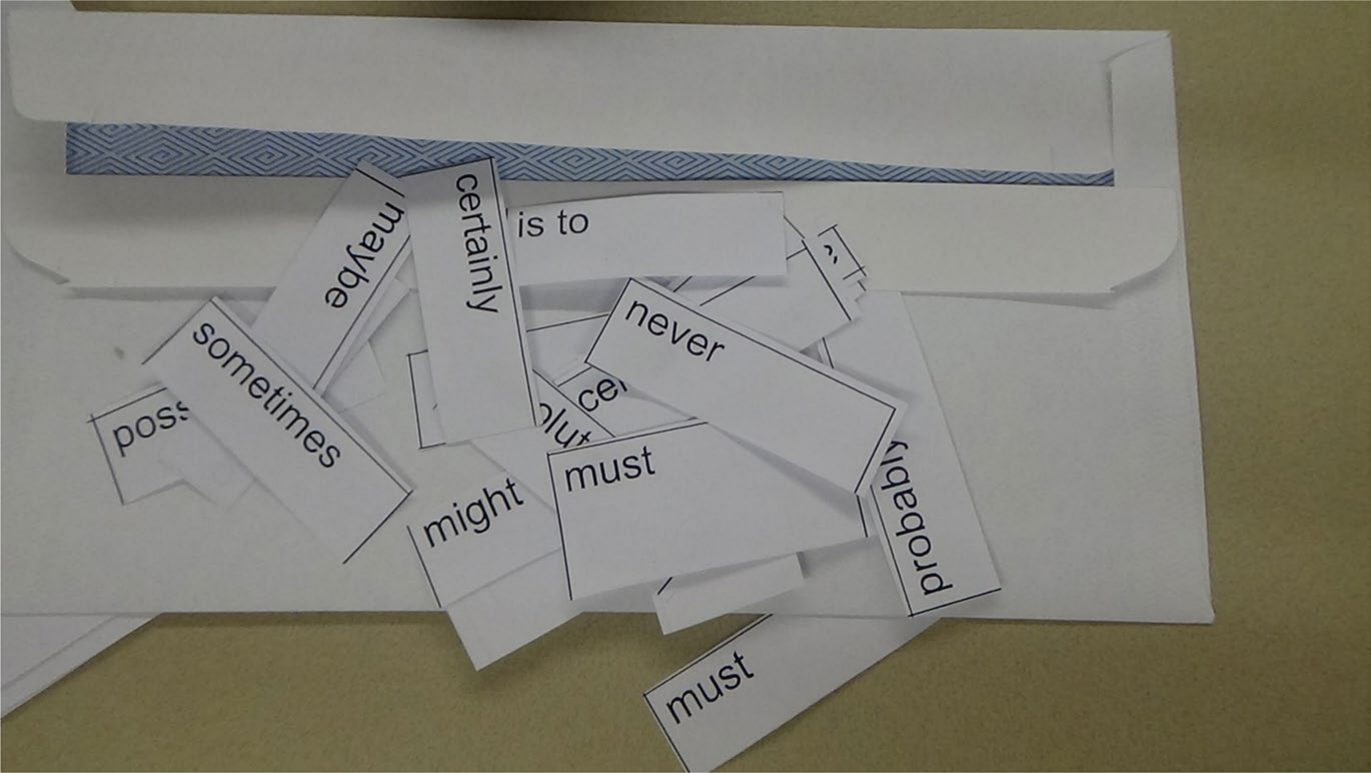
Modality collaborative activity

**Fig. 9:**
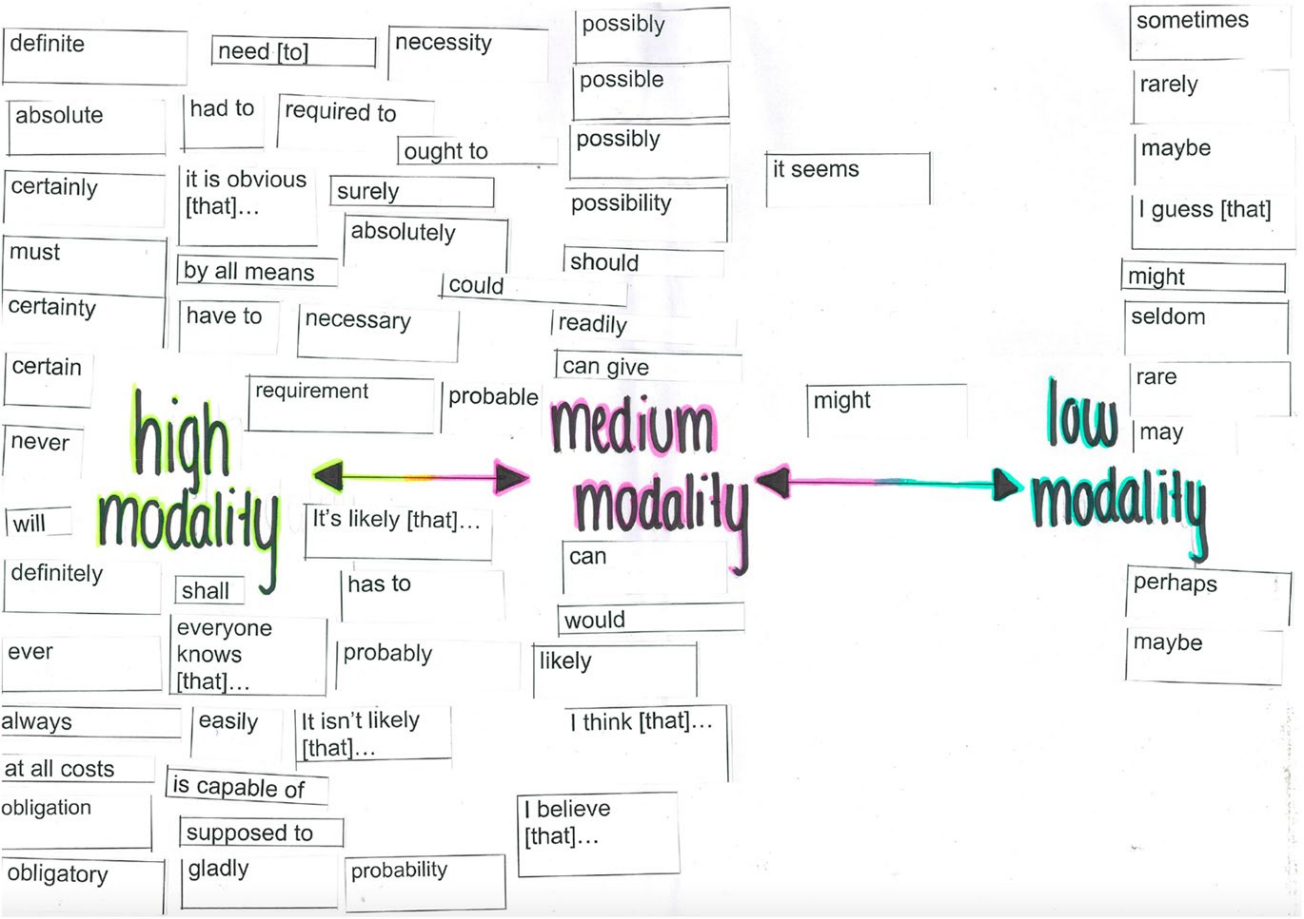
Modality map

**Fig. 10:**
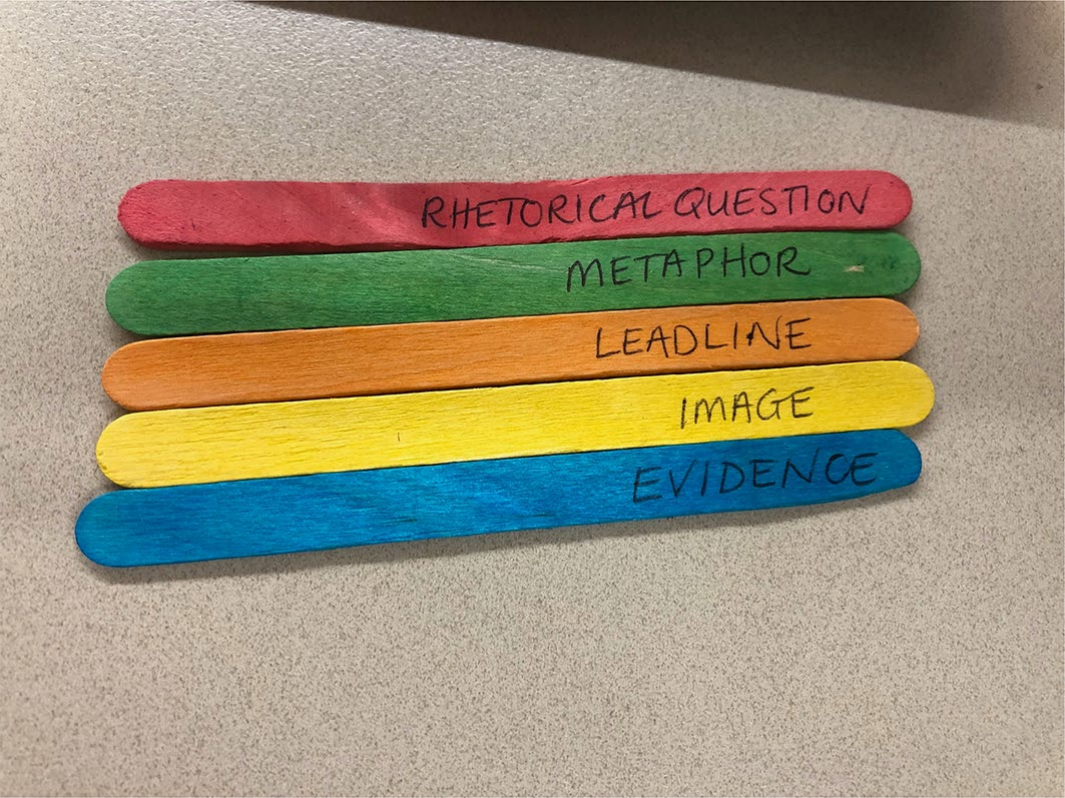
Paddlepop sticks

The first artefact uploaded comprised a cluster of items from one lesson demonstrating how students are taught to recognise and deploy variations in modality for effect. On the rubric for the feature article that they are working towards, modality pertains to just one of the skills-related criteria “discerning variations in vocabulary choices for impact”. Nevertheless, this teacher prioritises explicit teaching and approaches this through a series of stages that increase student independence. The lesson begins with direct instruction, followed by a guided activity, and then a collaborative learning activity. The learning intentions are written on the whiteboard: “students will define modality,…identify modal words, …discuss examples of modality, …collaborate in order to evaluate the modality of certain words and phrases”. A photocopied handout from a textbook includes a table of types of modal words: modal verbs, modal adverbs, modal adjectives, modal nouns, modal clauses and phrases; and examples of high, medium and low modality of each of these forms. The images that we have included here as exemplary artefacts are mobilised in a small group phase of the lesson. Envelopes containing (60+) cut out slips with modal words are distributed for a collaborative sorting activity (Fig. 8). Each group produces their own “modality map”, constructing a continuum of high to low modality words from the slips of paper (Fig. 9). The teacher explains “I model. I tell them everything and then I try to send them off to do it together”. Following the lesson, an online homework activity on the class Flipgrid site requires students to explain modality in their own words and provide original examples of high, medium and low modality. As they work towards their assessment task, a persuasive text in the form of a feature article, the teacher expects to see them deploying modality for effect (Fig. 10).

This image shows a homemade artefact comprising five labelled and coloured Paddlepop icypole sticks: red for *Rhetorical Question*, green for *Metaphor*, orange for *Leadline*, yellow for *Image*, blue for *Evidence*. These are a sample of the 22 or so visual prompts created by the teacher, listing language devices that are applicable to the feature articles they are writing. All of them have already been explicitly taught. They provide an independent checklist of language features that can be added to as students learn new language features through the year, or culled and used selectively for different genres of text. Even when the teacher had forgotten about these tools, the students would “come up and keep swapping around”, even keeping lists in their notebooks of those they had already used to review their drafts.

This teacher describes this approach to writing in terms of student responsibility and authenticity. These very competent students need to learn to take risks, aspire to authenticity and develop an individual “style” that will “carry them through”. A “good classroom” is one where students become more independent: “I feel happiest when I can hear the children talking about it themselves” in contrast to “when it’s just me talking and saying, you know, does that make sense and does that work—and they’re all polite and lovely that they’ll just nod, you know”. It is when the students are active and collaborative learners that the teacher feels that pedagogy has been effective: “When they are teaching each other is when I feel really happy about working”. The artefacts that were uploaded are tools that enable students as individuals and as a cohort to be become more self-reliant and independent writers.

## Discussion

The case studies we have developed in this paper are necessarily partial and unable to represent the breadth and complexity of pedagogy, even in these schools and classrooms. However, they suggest some of the common characteristics of English teaching. They oscillate between the creativity and individuality of voice that tends to be valued in progressivist or growth traditions, manifest in English as process writing, and the demands for textual evidence, analysis and argument required for literary criticism. These are different kinds of writing that we would expect students to master through their studies in English. Students are apprenticed into these traditions in their English classes and equipped through varying approaches to skill development, explicit teaching and rehearsal in low stakes contexts. Highly structured approaches to language and form are also evident. We would expect to find a focus on both creativity and structure in the teaching of writing in secondary English, with pedagogical attention extending beyond mechanistic naming of parts of speech or language features into how knowledge and understanding of them can facilitate their artful and purposeful manipulation in contexts of use to increase effectiveness of writing. It is clear also that teachers seek opportunities, within the varying constraints of their contexts, to express pedagogic, professional and collaborative agency (Chisholm et al. 2019). As we have noted, this sampling of artefacts is incomplete and the animation of artefacts can only happen effectively in pedagogical spaces that are shaped by the dynamic, responsive and performative arts of teaching.

None of the teaching artefacts offered to the research by teachers was explicitly directed towards NAPLAN, and none of them was collected close to the period of NAPLAN testing. However, all teachers talked about NAPLAN in their interviews. Their discussions of NAPLAN were not directly attached to the artefacts that we have discussed in the case study section. However, implicit references to NAPLAN can be ascribed to some of the pedagogical discussion in the previous section. Mention of a “low stakes” context for writing implies the “high stakes” context that is opposite to this—NAPLAN. Although whole school literacy approaches preceded NAPLAN, attention to “explicit teaching” and “direct instruction” has expanded through corporate support for selected professional learning programmes that are intended, amongst other effects, to improve NAPLAN results.^3^ The homogenising formulaic approaches to paragraphing signalled by acronyms like TEEL (or PEEL) are critiqued in writing research, but widespread in practice and perceived by many teachers to be beneficial to student persuasive writing in NAPLAN (McKnight 2020). Overall, the perception of NAPLAN as a measurement was that it is overstated, inaccurate or unrepresentative of the capabilities of students. Discussions related to NAPLAN and writing instruction concentrated on teacher concerns about rigidity of genre and text structures, the role of English teachers as literacy instructors and NAPLAN’s role in school planning.

School-wide concerns about declining writing results have prompted increased influence of school leaders in planning around NAPLAN. The extent to which teachers felt they needed to “teach to the test” was dependent on the nature of leadership in the school. Practices such as declaring “learning intentions” at the start of each lesson may be school or system-wide requirements. Some leaders call into question the experience and expertise of English teachers, whilst at the same time placing the burden of success in NAPLAN literacy on them. Some teachers raised questions around the need for direct instruction for specific marking criteria: “We’ve been told this year we have to explicitly teach… the marking criteria for NAPLAN… and it makes me a little twitchy”. Rather than teaching explicitly for NAPLAN, these teachers were focussed on “ongoing processes like unpacking the criteria and applying it to your work and having a writing process where you plan and write and refine”. Whilst one head of English would prefer “that whatever we were teaching is the good pedagogical practices for writing and skills transferred across to NAPLAN”, their approach has provoked “fights and conflict” with school leadership particularly about why students were not familiar with the various point scores for the NAPLAN marking criteria. She describes this interaction: “I said because we don’t teach them and I don’t care. What I wanted was a proper set of strategies about what they did in the first five minutes of getting the writing stimulus, because that’s a much more transferable skill”. On the other hand, given the opportunity to use NAPLAN productively, teachers were able to approach “NAPLAN data to look more longitudinally along what were kids at our school doing really well in their writing responses and what were the areas we were missing”. Using NAPLAN productively was perceived as allowing English teachers to direct their focus towards areas backed by data such as cohesion, rather than perceived concerns in the school such as spelling. “We didn’t look at the NAPLAN data and go ‘Here’s the crisis, we’ve got to address that’. It was the other way around”.

The position of teachers in the school varied in terms of how they were able to argue for or against demands of school executive in relation to literacy instruction. In the case above, the head of English was able to use their authority to advocate for a position that aligned with the school’s overall approach to explicit instruction whilst maintaining their position of not teaching to the test: “you’ve got to hold the line on what was good English teaching, what was good literacy teaching, to be able to put together a case for why we shouldn’t do NAPLAN practice in school”. For the teacher who sought to allow students to play with language and focussed on “what good writers do”, the lead up to NAPLAN presented a conflict. Overall, their intention was “to be brave and think about the big picture”; however, they recognised that “obviously there will be NAPLAN and I will probably have to do the things you have to do which will kill me inside”. The extent to which teachers respond to the expectations of planning their teaching year around the May testing period is apparent in how faculties sequence their teaching of genre. For example, “Term One is always persuasive and or narrative writing… most of the time it’s more just we are teaching the genre and teaching persuasive writing techniques to a text that we are starting”. The perceived role of English teachers as literacy experts and instructors is evident in the pressure experienced by those interviewed. The teachers were concerned to plan for writing instruction that develops a breadth of rich transferable skills and individual styles. However, in attempting to meet school-wide demands for literacy instruction and the perception of writing instruction as being “all the English teachers’ responsibility”, they felt pressured and targeted. NAPLAN results meant that “all of a sudden it became how can we teach more writing in English?” rather than a “school-wide approach” where literacy was every teacher’s responsibility.

That the genres privileged in the writing test are associated with English raised concerns about the rigidity of genre and text structures in the NAPLAN writing test and how this was reflected in expectations of writing pedagogy. Teachers expressed concerns about the rigid text structures students brought to high school from primary school, which made it difficult for them to work more freely and in more complex ways with language. One teacher found herself working against primary school habits of stories that would follow the structure of “Once upon a time, here’s the middle where something happens, here’s the end, it is very NAPLAN-ny narrative”. Although they recognised that this could be a necessary teaching strategy for primary students, they believed it was more important for secondary students to “really have a go at mucking around with writing”. However, once “NAPLAN comes around and you say, right now I want just want you to go back to that beginning, middle, end. And I want you to do all these things. And they’re like, but you said don’t do that”. The interference and imposition of expectations around teaching for NAPLAN results in responses that are “all the same” and demonstrate limited creativity. One teacher offered the opinion that a good score in NAPLAN could be interpreted as a teacher being “very good at teaching kids a formula. That isn’t the sign of a good teacher”. Teachers’ understanding of the need to adapt their practice in the moment—evident in their creation of artefacts designed to respond to particular demands or challenges of a class—is a reflection of the knowledge that students do not all learn in the same ways.

Regardless of the varied philosophies, beliefs and values expressed by the English teachers involved in the study, it is evident that concern for their students is what drives their teaching. Despite the disproportionate emphasis placed in NAPLAN by schools and governments, as one of our participants pointed out “at the end of the day, it is a small measure, and a not very accurate one. I don’t think it allows kids to show what they’re capable of”. Our findings about English teachers’ assessments of NAPLAN and its impacts in their schools and classrooms support those of other researchers outlined early in this paper (e.g. Carter et al. 2018; Cumming et al. 2018; Frawley and McLean Davies 2015; Simpson Reeves et al. 2018), reinforce the insights from the large-scale survey that preceded these case studies (Gannon 2019) and justify the deeper dive into pedagogy enabled by a qualitative design. The absence of a defining philosophy underlying NAPLAN disconnects it from the materialities and intricacies of classroom practice.

## Conclusion

The artefact-driven case studies that we have sketched in this paper suggest differing approaches to writing instruction that could prompt questions about which approach is more successful. However, if that question is still being answered by NAPLAN testing, success or otherwise might be beside the point whilst NAPLAN continues to obscure the need to accommodate and differentiate according to context. The attempt to provide a national standardised test and result relies on a form of testing that does not account for what teachers are attempting in their classrooms: engagement, individuality, explicit knowledge of language features, effective planning, editing and proofreading skills. Nor does it recognise the salience of teacher agency and its complex relationships to student learning (Chisholm et al. 2019). Overall, discussions with teachers highlighted the importance they placed on developing their students’ literary and literacy knowledge and practices.

Foregrounding teacher-generated artefacts that organise pedagogy has allowed insights into the materialities of practice, with discussions extending to teachers’ philosophies, beliefs, values and feelings about teaching writing, particularly in the context of NAPLAN. We did not find, or seek to describe, homogenous practices in the teaching of writing. Rather, we sought glimpses into the variety of pedagogical practice in secondary English classrooms. Furthermore, the case study at a distance approach that was developed for the study has proven to be surprisingly and unfortunately apt for these times. The crisis instigated by COVID-19 has had profound deleterious effects on all aspects of human life. It has, however, inadvertently interrupted the tenacious hold that a decade of NAPLAN testing has had on Australian schools. In this pause, significant reviews are underway into its impact; however, their potential impacts are difficult to gauge given the weight of evidence that has so far been disregarded by educational bureaucracies. Thus far, all that ACARA has committed to is a 1-year deferral of the transition to full online national delivery of NAPLAN.

## Footnotes

1 Initial discussions proposed a national study in partnership with all state ETAs; however, funding was sourced internally at Western Sydney University for a small pilot study in two states that required no financial contribution from the two state ETAs. ETA support was essential at all stages of the research, including recruitment of the participating case study teachers. We thank those teachers for sharing their practice and materials. 

2 The descriptive case studies have been included in a research report provided to AATE and its state member organisations, including ETAQ and TATE.

3 Such as John Hattie’s “visible learning” and Anita Archer’s “explicit instruction”.
